# Editorial: Innovations in teaching and learning: international approaches in developing teacher education and curriculum for the future

**DOI:** 10.3389/fpsyg.2024.1403661

**Published:** 2024-04-05

**Authors:** Markus Talvio, Marco Ferreira, Lawrence Meda

**Affiliations:** ^1^University of Helsinki, Helsinki, Finland; ^2^Sharjah Education Academy, Sharjah, United Arab Emirates; ^3^Instituto Superior de Educação e Ciências, ISEC Lisboa, Lisbon, Portugal; ^4^UIDEF, Instituto de Educação, Universidade de Lisboa, Lisbon, Portugal

**Keywords:** twenty-first century skills, positive psychology (PP), social and emotional learning (SEL), technology in learning, teacher professional development, innovation in learning, classroom dynamics, learning engagement

Over the past few years, educators worldwide have confronted a surge of unprecedented challenges in refining their instructional methods. Factors such as population mobility, an unstable labor market, and the rapid pace of globalization, as noted by the United Nations (2020), have contributed to this dynamic shift. Moreover, the education field undergoes continuous transformation, necessitating ongoing adaptations to redefine and enhance learning and teaching environments. Contemporary educational psychology, exemplified by movements like positive psychology, emphasizes aspects such as fostering belonging and collaborative knowledge creation. This calls upon teachers to prioritize facilitating their students' learning and wellbeing, thus creating positive learning environments over traditional teacher-centered approaches (Seligman, 2011; Csikszentmihalyi, 2014; Talvio and Lonka, 2021).

The advent of the digital revolution further challenges educators to embrace new educational paradigms and incorporate digital solutions into their pedagogical strategies. Indeed, the COVID-19 pandemic has accelerated this need, prompting teachers to innovate and provide optimal learning experiences (Lonka and Talvio, 2021). Teachers should be empowered to use their professional knowledge, competences, and expertise to deliver the curriculum successfully. A decisive commitment to teachers' continuous training becomes indispensable, namely through the strengthening of knowledge based on empirical evidence that can focus on a curriculum “adaptable, dynamic and collaborative” (Ferreira, 2023).

The primary objective of this Research Topic was to compile recent studies on pedagogy and educational psychology, focusing on innovations in teaching and learning worldwide. By gathering scientific insights from diverse stakeholders such as curriculum developers, practitioners, and researchers, the aim was to conceptualize, evaluate, and develop creative pedagogical concepts and practices suitable for the diverse contexts of twenty-first-century educational settings. In this Research Topic, we have strong international contribution covering countries in Europe, Asia, and the Middle East.

The Research Topic revolves around three core themes. The first theme focuses on *teacher-student interaction and classroom dynamics*. Martinsone and Žydžiūnaite delved into pivotal factors crucial for cultivating positive school climates and nurturing empathetic relationships with students. Their study aimed to grasp the needs of teachers in educator training, with the goal of equipping them with the requisite knowledge and skills to bolster their students' wellbeing. In their article, Rodríguez-Ferrer et al. investigated the effects of a game-based learning program on classroom environments and student engagement. Their findings provide compelling evidence of the benefits of employing game-based learning in high schools located within socially disadvantaged communities in Spain. Hojeij et al. explored the experiences of Emirati female preservice teachers with virtual classroom management. They underscored the significance of training in virtual learning technologies for both teachers and students to achieve satisfactory learning outcomes. The impact of feedforward on enhancing critical thinking and academic writing skills among pre-service teachers were examined by Baroudi et al.. Their research revealed that this teaching approach not only increased students' motivation to improve their performance in subsequent tasks but also elevated the quality of their work.

The second theme of the Research Topic focuses on *language learning and various teaching methods*. He and Oltra-Massuet examined whether advanced English as a Foreign Language (EFL) learners could reach a level of proficiency similar to that of native English speakers. They utilized the Elicited Oral Imitation Task test to assess learners' grammatical sensitivity and language production abilities in constructing English questions with refined grammatical errors. The study compared the performance of EFL participants with that of native speakers, revealing that advanced EFL students faced challenges in attaining implicit knowledge of English questions at the level of native speakers. Additionally, they observed a disparity between the language knowledge level and language production competence of EFL learners. In another study within this theme, Xu and Wang investigated how EFL student teachers transform their Pedagogical Content Knowledge (PCK) into Personal Practical Knowledge (PPK) within blended learning communities. The research findings shed light on the development of personal practical knowledge among EFL student teachers and its contribution to teacher education research and pedagogy.

The third theme of the Research Topic centers on *educational trends and teacher professional development*. Cao conducted a meta-analysis to assess the effectiveness of blended learning, taking into account factors like student performance and engagement. Their findings showed that while blended learning typically improved performance, attitudes, and achievements, it did not necessarily enhance student engagement in academic activities. Cui and Yin examined the impact of professional training on fostering teaching innovation among senior high school educators. They discuss their findings as a guide for developing more targeted and personalized teacher training programs, emphasizing the importance of autonomy for school administration and peer support in promoting teaching innovation. Lastly, Akcan et al. investigated teachers' perspectives on the implications of STEM education for the job market. Based on in-depth interviews with 32 teachers, they concluded that STEM education offered many benefits to learners, though some concerns were raised. The authors also provide suggestions for the future of STEM education.

## Author contributions

MT: Writing – original draft, Writing – review & editing. MF: Writing – original draft, Writing – review & editing. LM: Writing – original draft, Writing – review & editing.
